# γδ+ T-Cells Is a Useful Biomarker for the Differential Diagnosis between Celiac Disease and Non-Celiac Gluten Sensitivity in Patients under Gluten Free Diet

**DOI:** 10.3390/nu16142294

**Published:** 2024-07-17

**Authors:** Albert Martín-Cardona, Anna Carrasco, Beatriz Arau, Judith Vidal, Eva Tristán, Carme Ferrer, Gerardo Gonzalez-Puglia, Natàlia Pallarès, Cristian Tebé, Sergio Farrais, Concepción Núñez, Fernando Fernández-Bañares, Maria Esteve

**Affiliations:** 1 Gastroenterology Department, Hospital Universitari Mútua Terrassa, University of Barcelona, 08221 Terrassa, Spain; albertmartin@mutuaterrassa.cat (A.M.-C.); anna.carrasco.garcia@gmail.com (A.C.); beatrizarau@mutuaterrassa.es (B.A.); etrislo23@gmail.com (E.T.); gerardgonzalezpuglia@gmail.com (G.G.-P.); 2 Centro de Investigación Biomédica en Red de Enfermedades Hepáticas y Digestivas (CIBERehd), Instituto de Salud Carlos III, 28029 Madrid, Spain; 3 Department of Flow Cytometry, Catlab, 08232 Viladecavalls, Spain; jvidal@catlab.cat; 4 Pathology Department, Hospital Universitari Mútua Terrassa, University of Barcelona, 08221 Terrassa, Spain; carmeferrer@mutuaterrassa.es; 5 Biostatistics Support and Research Unit, Germans Trias i Pujol Research Institute and Hospital (IGTP), 08916 Badalona, Spain; npallares@igtp.cat (N.P.); ctebe@igtp.cat (C.T.); 6 Gastroenterology Department, Hospital Universitario Fundación Jiménez Díaz, 28040 Madrid, Spain; farrais84@gmail.com; 7 Laboratorio de Investigación en Genética de Enfermedades Complejas, Hospital Clínico San Carlos, Instituto de Investigación Sanitaria del Hospital Clínico San Carlos (IdISSC), 28040 Madrid, Spain; conchita.npardo@gmail.com

**Keywords:** celiac disease, non-celiac gluten sensitivity, gluten-free diet, intraepithelial lymphogram, γδ T cells, flow cytometry

## Abstract

Background: The differential diagnosis between patients with celiac disease (CD) and non-celiac gluten sensitivity (NCGS) is difficult when a gluten-free diet (GFD) has been initiated before the diagnostic work-up. Isolated increases in TCRγδ+ and celiac lymphogram (increased TCRγδ+ plus decreased CD3−) may enable differential diagnosis in this challenging clinical setting. This study evaluated: (1) the accuracy of %TCRγδ+ and celiac lymphogram for diagnosing CD before and after GFD and for differentiation with NCGS; (2) TCRγδ+ kinetics at baseline and after starting GFD in both CD and NCGS. Methods: The inclusion criteria were patients with CD (n = 104), NCGS (n = 37), and healthy volunteers (n = 18). An intestinal biopsy for intraepithelial lymphogram by flow cytometry was performed at baseline and after GFD. The optimal cutoff for CD diagnostic accuracy was established by maximizing the Youden index and via logistic regression. Results: %TCRγδ+ showed better diagnostic accuracy than celiac lymphogram for identifying CD before and after GFD initiation. With a cutoff > 13.31, the accuracy for diagnosing CD in patients under GFD was 0.88 [0.80–0.93], whereas the accuracy for diagnosing NCGS (%TCRγδ+ ≤ 13.31) was 0.84 [0.76–0.89]. The percentage of TCRγδ+ cells showed differential kinetics between CD (baseline 22.7% [IQR, 16.4–33.6] vs. after GFD 26.4% [IQR, 17.8–36.8]; *p* = 0.026) and NCGS (baseline 9.4% [IQR, 4.1–14.6] vs. after GFD 6.4% [IQR, 3.2–11]; *p* = 0.022). Conclusion: TCRγδ+ T cell assessment accurately diagnoses CD before and after a GFD. Increased TCRγδ+ was maintained in the long term after GFD in CD but not in NCGS. Altogether, this suggests the potential usefulness of this marker for the differential diagnosis of these two entities in patients on a GFD.

## 1. Introduction

Celiac disease (CD) involves a systemic autoimmune process resulting from a permanent intolerance to gluten and occurs in genetically susceptible individuals [1,2]. Non-celiac gluten sensitivity (NCGS) is a syndrome characterized by intestinal and extraintestinal symptoms related to the ingestion of gluten-containing food in subjects with normal duodenal mucosa, where either CD or wheat allergy has previously been ruled out while the patient is still on a gluten-containing diet (GCD). The prevalence of CD in Western countries is around 0.6% histologically confirmed and 1% in serological screening of the general population. In contrast, knowing the true prevalence of NCGS is difficult and is generally based on self-reported symptoms. Probably for that reason, the prevalence is highly variable, ranging from 0.5% to 13% in Western population.

From a pathophysiological perspective, these diseases are very different. In CD, gluten peptides resistant to degradation cross the intestinal barrier increasing intestinal permeability. In the lamina propria, tissue transglutaminase (tTG) deamidates these peptides, increasing their affinity for HLA-DQ2/DQ8 on antigen-presenting cells (APCs). APCs present these peptides to CD4+ T lymphocytes, activating them and triggering a cytokine-mediated inflammatory response. This induces the production of anti-tTG, anti-gliadin, and anti-endomysium antibodies, and activates cytotoxic CD8+ T lymphocytes, which damage enterocytes. Chronic inflammation results in intestinal enteropathy, from lymphocytic enteritis to atrophy, reducing the intestinal absorption surface and causing nutrient malabsorption [1,2]. By contrast, the pathophysiology of NCGS is poorly understood and may involve various triggers similar to those in CD and irritable bowel syndrome [3]. The main trigger is dietary gluten, which can lead to immune-mediated and/or non-immune-mediated responses. Unlike CD, T cell involvement is not evident in NCGS, suggesting that may involve predominantly an innate immune response, possibly through toll-like receptors (e.g., TLR-1, TLR-2). Changes in the gut microbiome due to gluten consumption might also influence NCGS. Recent data indicate that TLR4 may play a role in NCGS pathogenesis by transducing the effect of gliadin through the MYD88 adaptor protein, leading to increased expression of pro-inflammatory cytokines and systemic immune activation [1,4,5].

The digestive symptoms of both entities can be indistinguishable, and the treatment is the same: a gluten-free diet (GFD). CD diagnosis should be made in patients who are following a normal GCD and is based on celiac serology and histopathological changes in the small intestinal mucosa [1], whereas in NCGS the duodenal mucosa is normal and celiac serology is absent [1]. However, many patients seek to rule out CD after having started a GFD. In this scenario, diagnosing CD and the differential diagnosis with NCGS remains a challenge, as most CD-associated changes revert after gluten withdrawal. This is a very frequent situation in clinical practice and currently, a gluten challenge test is recommended. However, the way to do the challenge is not well standardized. It is not clear the dose of gluten that must be administered, the form of administration (whole bread, capsules, etc.) or the duration of the trial. In addition, many patients do not accept it because it is poorly tolerated. Current diagnostic guidelines [1] consider differentiating both entities to be very important, since the complications (including malabsorption and intestinal lymphoma) greatly differ, and the level of adherence required for the GFD has to be much stricter in CD than in NCGS.

An essential finding of CD is the increased number of total intraepithelial lymphocytes (IELs) in the duodenal mucosa, characterized by an expansion of γδ+ and CD8+ IELs coupled to a decrease in CD3− IELs [6,7]. Flow cytometry allows concomitant and accurate quantification of these two cell subsets, resulting in a celiac lymphogram (increased %TCRγδ+ plus decreased %CD3− cells), with high diagnostic accuracy for CD [8,9]. In addition, flow cytometry represents a simple, fast, and inexpensive tool that may strengthen the diagnosis of CD in cases that are not straightforward [9].

The increase in the TCRγδ+ subset appears to be a permanent feature of CD even with a GFD [8,9,10], which opens up the possibility of using this subset as a diagnostic tool in patients currently following a GFD, without the need for a gluten challenge. Flow cytometry has been applied in several studies focused on this topic with promising results, but the studies were performed on small samples of patients with follow-up time after GFD initiation rarely described [6,8,9,11,12,13]. In addition, changes in mean values before versus after the diet were reported in independent samples from unrelated groups [12,13,14,15,16,17]. Whether the TCRγδ+ subset is featured in the NCGS after GFD initiation remains unknown [4,5].

Thus, the aims of this study were to evaluate (1) the accuracy of TCRγδ+ for CD diagnosis before and after GFD initiation and to ascertain whether it is useful for differential diagnosis from NCGS and (2) TCRγδ+ and CD3− kinetics at baseline and in the long-term follow-up after starting a GFD in both CD and NCGS.

## 2. Materials and Methods

### 2.1. Study Design, Definitions, Patients and Controls

**Figure 1:**
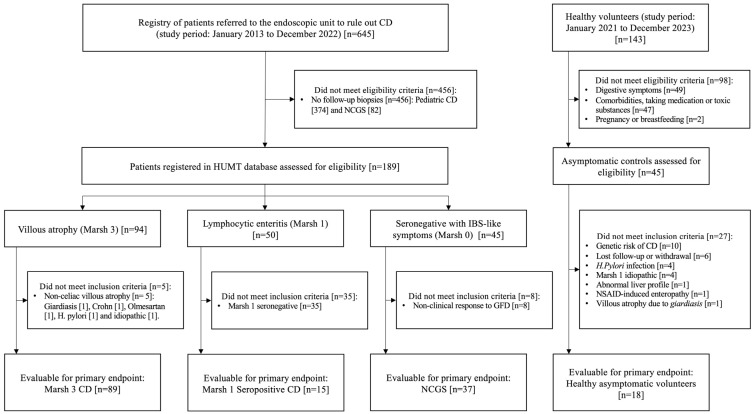
Study flow chart. Abbreviations: HUMT: Hospital Universitari Mútua Terrassa; GFD, gluten-free diet; IBS: irritable bowel syndrome; NSAID: nonsteroidal anti-inflammatory drug; CD, celiac disease; NCGS, non-celiac gluten sensitivity.

This study was designed to assess the accuracy of TCRγδ+ T cells for the diagnosis of CD and NCGS. Patients were identified from a prospective registry (January 2013 to December 2022) representing a database of patients referred for a celiac lymphogram to rule out CD. Patients with CD and with NCGS were included, with the latter being the disease control group (Figure 1). The inclusion criteria were (1) being on a GFD for at least one year and (2) undergoing a follow-up intestinal biopsy to assess the evolution of the intraepithelial lymphogram. The exclusion criteria were refusal to participate in the registry, pregnancy and pre-existing severe comorbidities, use of nonsteroidal anti-inflammatory drugs or olmesartan, Crohn’s disease, autoimmune disease-associated enteropathy, collagenous sprue, microscopic colitis, lymphocytic enteritis due to intestinal parasitosis or *Helicobacter pylori*, other enteropathies, and selective IgA deficiency. Investigators reviewed the medical records of all patients to ensure that they fulfilled the inclusion/exclusion criteria and to double-check the completeness and accuracy of the data. Clinical, serological, and histological responses to a GFD were retrospectively reviewed.

In all patients, we performed celiac serology, HLA-DQ genotyping, duodenal biopsy for histopathology, and IEL subpopulation analysis. In our department, routine follow-up biopsies are performed in adult CD patients at least 1 year after starting a GFD to assure histological remission. This strategy is based on the high frequency of persistent villous atrophy (VA) in adults on an apparently strict GFD despite being in clinical and serological remission [18].

CD diagnosis was based on the 2019 guidelines of the European Society for the Study of CD [1], as follows: 1. the presence of clinical symptoms of the CD spectrum or pertaining to a risk group; 2. the presence of compatible histology; 3. positive celiac serology; 4. a permissive HLA-DQ genotype and a clinical and serological response to a GFD in patients with Marsh 1. Seronegative patients with CD were diagnosed according to the recent Paris consensus criteria [19] as follows: 1. exclusion of all the other causes of VA; 2. permissive HLA-DQ genotype, and 3. sustained clinical and histological response to a GFD.

NCGS diagnosis was based on the 2019 guidelines of the European Society for the Study of CD and other gluten-related disorders [1] and was based on the following criteria: 1. the presence of irritable bowel syndrome-like symptoms and extraintestinal manifestations occurring after gluten ingestion, improving rapidly with a GFD; 2. a normal histological study (Marsh 0, ≤25% IELs); 3. negative celiac serology.

The healthy controls were prospectively recruited volunteers. They were strictly asymptomatic (assessed by a questionnaire of symptoms, available in the Supplementary Materials) and had normal duodenal mucosa without infections (*Helicobacter pylori*, parasites) or other organic digestive diseases (neoplastic or inflammatory). Subjects who had serious comorbidities, who were pregnant, who consumed tobacco or alcohol, or who were taking medication were excluded. To completely rule out CD, negative serology was mandatory, all alleles of the HLA-DQ were negative, and there had to be no history of first- or second-degree relatives of CD patients. All data needed from healthy controls were extracted from a larger ongoing study which involves evaluating many lymphocyte subpopulations in healthy subjects.

### 2.2. Duodenal Sample Collection

Biopsy samples were obtained using 2.8 mm biopsy forceps (Radial Jaw 4, Boston Scientific, Marlborough, MA, USA). The volume of the biopsies ranges between 2 to 3 mm³ depending on the depth of the biopsy. Four endoscopic biopsies were taken from the second-third portion of the duodenum and one from the duodenal bulb and processed using hematoxylin/eosin staining and CD3 immunophenotyping. The lymphocyte count was determined as previously described [20,21]. Two endoscopic biopsies from the antrum were also obtained to investigate *Helicobacter pylori* in all patients and controls.

### 2.3. Histopathological Assessment

The Marsh-Oberhuber classification [22] divides duodenal lesions into 3 mains categories (Marsh 1, 2, and 3) and 3 subcategories (3a, 3b, and 3c). Marsh 0 is defined as nor-mal duodenal mucosa. Marsh 1 lesions (lymphocytic enteritis) is defined by the presence of increased intraepithelial lymphocytes (IEL) by 25 or more IELs per 100 epithelial cells along with normal villous architecture [20,23,24]. Marsh 2 by the presence of crypt hyperplasia, and Marsh 3 by the presence of villous atrophy (VA) (3a partial atrophy, 3b subtotal atrophy, and 3c total atrophy). To simplify the analysis, all samples have been classified as Marsh 0, Marsh 1, or Marsh 3.

In patients with Marsh 3 scores, histological remission was considered when the follow-up biopsies showed Marsh 0 (<25% IELs) or the Marsh I category [25]. In patients with Marsh 1 at baseline, histological remission was considered when the follow-up biopsy was normal (IEL count <25%) or there was a reduction ≥50% from baseline.

### 2.4. Celiac Serology

Serum IgA-tissue transglutaminase antibody (anti-tTG2) was analyzed using a quantitative automated ELISA detection kit (Elia CelikeyTM, PhadiaAB, Freiburg, Germany) with recombinant human TG2 as the antigen. The cutoff of positivity established by the provider was 8 U/mL. However, since 99% of the general population in our location had values of anti-tTG2 < 2 U/mL [26], values between 2–8 U/mL (borderline) were considered positive if confirmed by positive serum IgA anti-endomysium antibodies (EmA). Values ≤ 30 U/mL were considered positive at low titers, and values >30 U/mL were considered positive at high titers. EmA was performed in all patients with either positive low titers or borderline anti-tTG2 by indirect immunofluorescence assay in serum samples at a 1:5 dilution (commercial sections of monkey distal esophagus; BioMedical Diagnostics, Marne-la-Vallée, France). Total serum IgA was measured using rate nephelometry (BN II, Siemens Healthcare Diagnostics SL, Marburg, Germany).

The follow-up of GFD adherence was carried out by a specialized dietician, and urine immunogenic gluten peptides were performed twice a year [27].

### 2.5. HLA-DQ Genotyping

Genomic DNA from whole blood was purified using a commercial QIAamp DNA Blood Mini Kit (Qiagen, Düsseldorf, Germany). A commercial reverse hybridization kit for the detection of the CD heterodimers HLA-DQ2.5 (HLA-DQA1*05:01/*05:05, HLA-DQB1*02:01/*02:02) and HLA-DQ8 (HLA-DQA1*03, HLA-DQB1*03:02) was used (GenID, GMBH, Strasburg, Germany). The HLA-DQ2.5 haplotype is present in approximately 24% of healthy controls and 90% of CD patients in our geographical area. Permissive DQ genotyping was performed according to previous recommendations [28].

### 2.6. Intestinal Lymphocyte Isolation and Quantification by Flow Cytometry

We performed IEL flow cytometry by obtaining an additional duodenal biopsy from the second portion of the duodenum, which was immediately processed as previously described [8,14,21,29,30]. No adverse events occurred in the collection of duodenal biopsies. The samples for the study of lymphocyte subpopulations were collected in complete culture medium, which was sterile Advanced RPMI supplemented with 2% FBS, 1% antibiotic-antimycotic 100× (10,000 U/mL penicillin, 10,000 µg/mL streptomycin, 25 µg/mL amphotericin B) to prevent cell culture contamination, and 1% L-glutamine 200 mM for cell culture supplementation; both reagents were obtained from Gibco (Refs. 11570486 and 11500626, Thermo Fisher Scientific Inc., Waltham, MA, USA). IELs were isolated by gentle rotation in an orbital shaker at 12 rpm for 90 min in a solution of 1 mM DTT and 1 mM EDTA in 10% FBS Hanks balanced salt solution (HBSS) at room temperature. After two washes with HBSS (10 min, 300 g), the IELs were stained with previously titrated amounts of directly labeled antibodies for 15 min at room temperature. The antibodies used for IEL staining were anti-CD45-APC (clone 2D1), anti-CD3−PerCP (clone SK7), anti-CD103-FITC (clone BerACT8), and anti-TCR γδ-PE (clone 11F2), all from BD Biosciences, Franklin Lakes, NJ, USA. Single-cell suspensions were acquired by an 8-color digital FACSCanto II Flow Cytometry System (BD Bioscience, San Jose, CA, USA), and the data were analyzed with BD FACSDiva v9.0 software (BD Bioscience). The cell counts yielded after digestion of the recovered cell number per biopsy were made with a hemocytometer and trypan blue exclusion. The average number of recovered intraepithelial lymphocytes from one fresh biopsy was 353,258 ± 13,841 (270,773 ± 22,503 in patients with atrophy and 398,803 ± 24,403 in those without atrophy) [21]. Since the obtained cell suspension always allows for a proper flow cytometry staining (minimum 100,000 events) the cell counts were not systematically performed in all samples. All obtained lymphocytes after digestion were stained and evaluated by flow cytometry. The results were obtained after 3 to 4 h and are expressed as percentages of bright CD45 staining and a low sideward scatter gate. Live IELs were gated on a CD45 and low scatter basis, and intraepithelial origin was confirmed with CD103+ staining (≥85%). The flow cytometry photomultiplier tube voltages and compensation values were manually adjusted using single-stained samples. This protocol was validated internally and accredited by the “Entidad Nacional de Acreditación (ENAC)” according to the UNE-EN ISO 15189:2013 (Reference 989/LE1956) regulation, which provides guidance to clinical laboratories.

Based on the combination of TCRγδ+ and CD3− IEL values, the four lymphogram patterns were established [9]: (1) normal; (2) isolated decrease in CD3− cells; (3) isolated increase in TCRγδ+ cells; and (4) celiac lymphogram (increase in TCRγδ+ cells plus decrease in CD3− cells).

### 2.7. Statistical Analysis

To define cohort characteristics, categorical variables are presented as the number of patients and percentages, while continuous variables are presented as the mean and standard deviation (SD) or median and interquartile range (IQR). The paired Wilcoxon signed rank test (continuous variables) and McNemar’s test (categorical variables) were used to compare the median percentages of TCRγδ+ cells and CD3− cells in the CD patients and NCGS at baseline and at the end of the study. The chi-square test and Fisher’s test were used to determine the associations between persistent atrophy and positive serology with dichotomized %TCRγδ.

To compute the best cutoff value that separates %TCRγδ+ cells and celiac lymphogram between CD and healthy volunteers (gold standard), two methods were used. With the first method, the best cutoff was selected according to the Youden index. The second method consisted of running through all the possible values of the %TCRγδ+, dichotomizing the %TCRγδ+ variable according to each value, and calculating the odds ratio (OR) with a logistic regression model. The best cutoff value selected was associated with the smallest *p*-value of the variable in the model. With these two cutoffs, we dichotomized the variable into two groups (positive and negative). Sensitivity, specificity, positive predictive value, and negative predictive value were calculated for both cutoffs to describe the accuracy of each cutoff in detecting the outcome.

All analyses were performed with a two-sided significance level of 0.05 using R software version 4.3.0 (https://www.r-project.org/). The main R packages used for data management and analysis were ThresholdROC version 2.9.4 and epiR version 2.0.75 (https://cran.r-project.org/package=epiR).

## 3. Results

### 3.1. Baseline Characteristics of the Study Population

A total of 159 subjects were included and categorized as follows: 104 patients with CD (89 Marsh 3, 15 Marsh 1 seropositive), 37 with NCGS, and 18 healthy volunteers (Table 1).

### 3.2. Clinical, Histological, and Serological Evolution of Patients at Baseline and after a GFD

A follow-up biopsy was performed for all patients after a median of 2 years (IQR, 2–3) after the start of the GFD (Table 2). Eighty-eight out of 104 patients with CD (84.6%) were in clinical remission at the time of follow-up biopsy. Forty-three out of 104 patients (41.3%) had a normal duodenal mucosa, 45 (43.3%) had a Marsh 1 lesion, and only 16 (17.9%) had persistent VA. The IELs of patients in Marsh 1 significantly decreased (14 with histological remission and 1 with no response). Overall, histological remission occurred in 87 of 104 patients (83.6%). Celiac serology remained positive in 21 patients (20.2%), mostly with low or borderline titers.

All patients with NCGS had negative serology. The duodenal mucosa was normal at baseline (Marsh 0, ≤25% IELs) and remained normal during the follow-up.

### 3.3. Diagnostic Accuracy of γδ T Cells and Celiac Lymphogram for Identifying Patients with CD with or without Gluten Intake and the Differential Diagnosis with NCGS

We first performed an exploratory analysis to determine the best cutoff value to distinguish patients with CD from healthy subjects both at baseline (with a GCD) and at the final evaluation (with a GFD) (Table 3). For that purpose, we used combinations of different cutoff values obtained by using the Youden index and a logistic model for both isolated TCRγδ+ cells and the celiac lymphogram (TCRγδ+ and CD3−IELs).

The isolated %TCRγδ+ > 8.64 [logistic model] had the best diagnostic performance at baseline (accuracy 0.93) and at final evaluation (accuracy 0.90) for CD diagnosis. However, with this cutoff, the specificity was only 0.78. By contrast, the celiac lymphogram showed a better specificity while maintaining a good accuracy for CD diagnosis, but only before a GFD initiation at baseline (cutoff values for CD diagnosis [logistic model]: %TCRγδ+ > 8.64 and %CD3− ≤ 16.3; sensitivity 0.91 [0.84, 0.96]; specificity 0.94 [0.71, 1.00]; accuracy 0.92 [0.85, 0.96]).

**Figure 2:**
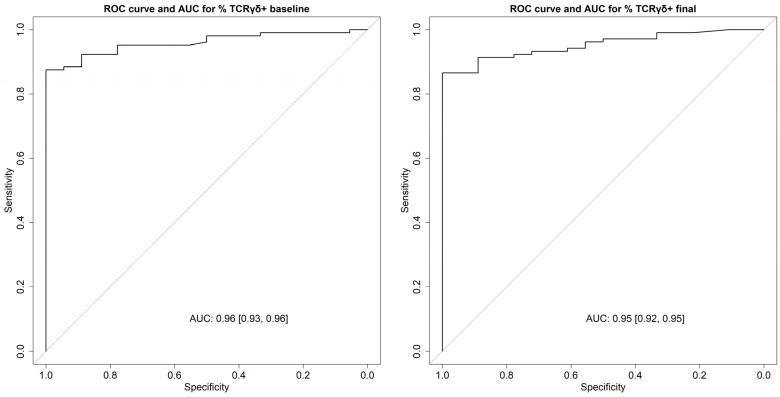
ROC curve to assess the accuracy of the TCRγδ+ IEL for identifying patients with celiac disease (gold standard healthy individuals). Abbreviations: AUC: area under the curve; ROC: receiver operating characteristic; TCR: T-cell receptor.

Since we were looking for the highest diagnostic specificity for CD diagnosis that provided us with a good differential diagnosis with NCGS, especially after GFD initiation, we chose the cutoff values of isolated %TCRγδ+ cells obtained by using the Youden index. With this method, the optimal cutoff points at baseline and final evaluation were 12.91 and 13.31 (accuracy for CD diagnosis: 0.89 and 0.88, respectively). Figure 2 shows the ROC curve for identifying CD patients (baseline and final).

Therefore, to establish the differential diagnosis between CD and NCGS only %TCRγδ+ cells were used and applied to the final sample with GFD, which is the setting with diagnostic uncertainty. The accuracy of the percentage of TCRγδ+ cells ≤ 13.31 to rule out CD in patients with NCGS disclosed the following results: sensitivity 0.86 [0.77, 0.91]; specificity 0.78 [0.61, 0.90]; positive predictive value 0.92 [0.84, 0.96]; negative predictive value 0.66 [0.50, 0.80]; accuracy 0.84 [0.76, 0.89].

### 3.4. TCRγδ+ Kinetics at Baseline and in the Long Term under a GFD in Patients with CD and NCGS

**Figure 3:**
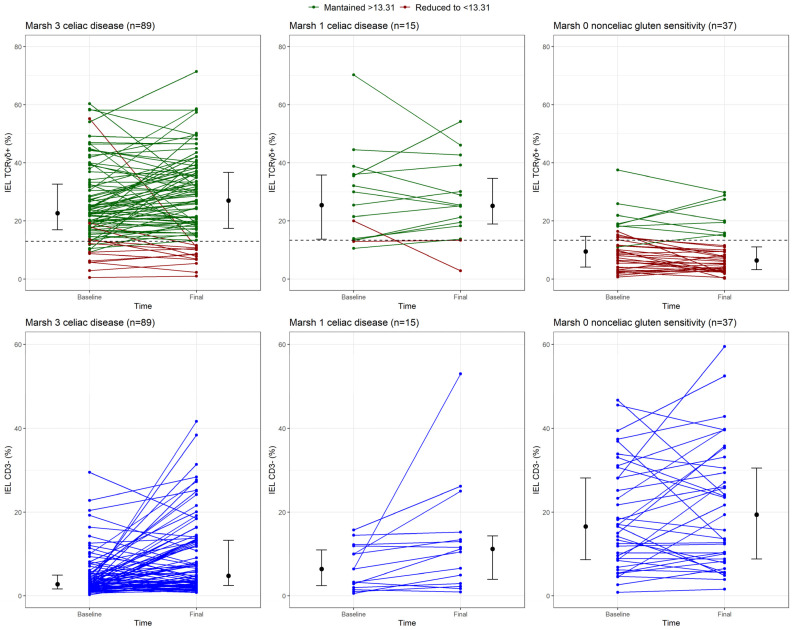
Median with interquartile range and dot and line diagrams describing the evolution of TCRγδ+ (green lines) and CD3− cells (blue lines) before and after a gluten-free diet (GFD) in patients with celiac Marsh 3, celiac Marsh 1, and non-celiac gluten sensitivity (NCGS). Black dotted line indicates the cutoff point for TCRγδ+ cells at 13.31%. Red lines indicate patients with nonpersistence of increased γδ T-subsets after a GFD (final TCRγδ+ cells ≤ 13.31%). Abbreviations: TCR: T-cell receptor; IEL: intraepithelial lymphocyte.

Figure 3 shows the changes in the percentages of TCRγδ+ and CD3− cells at baseline and at the final evaluation. The median percentage of TCRγδ+ cells was significantly greater in patients with Marsh 3 who received a GFD compared to baseline (*p* = 0.020), whereas the percentage stayed high in patients with Marsh 1 (*p* = 0.999). No significant differences were found between patients with Marsh 3 and those with Marsh 1, either at baseline or at the final assessment. Overall, 86.5% of patients with CD had increased percentages of TCRγδ+ cells despite receiving a GFD. The duration of GFD was 1 year in 19 patients, 2–3 years in 61 (median, 2 years; IQR 2–3) and ˃3 years in 24 (median, 5 years; IQR, 4–6). An increase in the percentage of TCRγδ+ cells was observed in 94.7%, 85.2%, and 83.3% of patients in each time subgroup, respectively. There was no relationship between persistent atrophy or positive serology and a %TCRγδ+ > 13.31 at the final evaluation with a GFD (chi-square *p* value = 0.240 for persistent atrophy and *p* value = 1.000 for positive serology).

**Figure 4:**
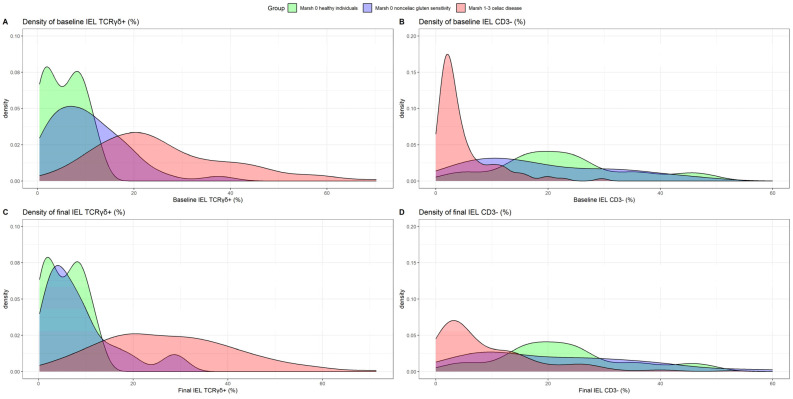
Baseline and final distributions of the percentage of TCRγδ+ cells (**A**,**C**) and percentage of CD3− cells (**B**,**D**) in the three groups: Marsh 0 healthy individuals, Marsh 0 non-celiac gluten sensitivity patients and Marsh 1–3 celiac disease patients. Abbreviations: TCR: T-cell receptor; IEL: intraepithelial lymphocyte.

Differences in TCRγδ+ density between patients with NCGS and with CD were observed (Figure 4). At baseline, significant differences were observed between CD (median %TCRγδ+ 22.66 [IQR 16.41–33.56]), NCGS (9.43 [4.10–14.66]) and healthy subjects (5.75 [1.80–8.59]); *p* < 0.001. The median values for NCGS patients decreased further in the final sample (6.40 [3.20; 11.00] vs. baseline; *p* = 0.022), resembling those of healthy controls. In fact, in Figure 4C, an almost complete overlap in the density of %TCRγδ+ cells may be observed between healthy controls and NCGS at final evaluation, which is well differentiated from CD. However, 12 patients with NCGSs at baseline (32.43%) and 8 (21.62%) with GFDs during follow-up had a %TCRγδ+ > 13.31. The clinical characteristics of these patients are detailed in Table 4.

**Figure 5:**
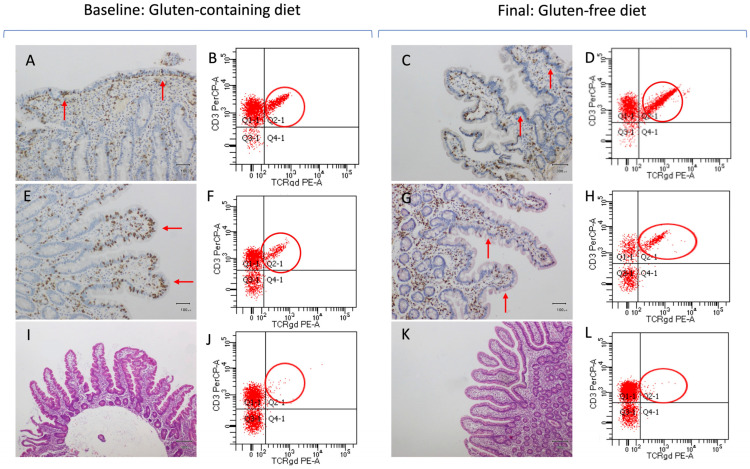
Pathological and intestinal cytometry images illustrating one case of three following categories in the study: the first row shows a patient with Marsh 3 celiac disease (**A**–**D**), the second row a patient with Marsh 1 celiac disease (**E**–**H**), and the third row a patient with non-celiac gluten sensitivity (**I**–**L**). The first two columns (**A**,**B**,**E**,**F**,**I**,**J**) show the images of patients on a gluten-containing diet, and the third and fourth columns (**C**,**D**,**G**,**H**,**K**,**L**) show the images of patients on a gluten-free diet. (**A**) Immunohistochemical staining of CD3 lymphocytes in duodenal biopsies with atrophy (Marsh 3) (orig. mag. x20). The red arrows indicate the accumulation of CD3 lymphocytes in the atrophic mucosa. (**B**) Intestinal cytometry panel showing increased γδ T-subsets (red circles) with GCD in a sample of duodenal atrophy (Marsh 3). (**C**) Immunohistochemical staining of CD3 lymphocytes in the recovered duodenal mucosa (Marsh 0) with a GFD (original magnification x20). The red arrows indicate the decrease of CD3 lymphocytes in the duodenal villi (orig. mag. x20). (**D**) Intestinal cytometry panel showing persistent increase of γδ T-subsets (red circles) with a GFD in a sample of recovered duodenal mucosa (Marsh 0). (**E**) Immunohistochemical staining of CD3 lymphocytes in duodenal biopsies with lymphocytic enteritis (Marsh 1) (orig. mag. x20). The red arrows indicate the accumulation of CD3 lymphocytes in the duodenal mucosa. (**F**) Intestinal cytometry panel showing in-creased γδ T-subsets (red circles) with GCD in a sample of lymphocytic enteritis (Marsh 1). (**G**) Immunohistochemical staining of CD3 lymphocytes in the recovered duodenal mucosa (Marsh 0) with a GFD (orig. mag.x20). The red arrows indicate the decrease of CD3 lymphocytes in the duodenal villi. (**H**) Intestinal cytometry panel showing persistent in-crease of γδ T-subsets (red circles) with a GFD in a sample of recovered duodenal mucosa (Marsh 0). (**I**) H&E staining of normal duodenal biopsies from a patient with NCGS (orig. mag. x4). (**J**) Intestinal cytometry panel showing low values of γδ T-subsets with a GCD in a sample of normal duodenal mucosa (Marsh 0) from a patient with NCGS. (**K**) H&E staining showing persistence of normal duodenal biopsies from a patient with NCGS (orig. mag. x4). (**L**) Intestinal cytometry panel showing persistent low values of γδ T-subsets with a GFD in a sample of normal duodenal mucosa (Marsh 0) from a patient with NCGS. Abbreviations: GFD: Gluten-free diet; GCD: Gluten-containing diet; H&E: Hematoxylin and eosin; orig. mag.: original magnification; NCGS: Non-celiac gluten sensitivity.

As previously mentioned, the accuracy of the celiac lymphogram (increased TCRγδ+ cells plus decreased CD3− cells) as a CD diagnostic marker in patients receiving a GFD is insufficient because the %CD3− significantly increased after GFD initiation compared to that at baseline (*p* < 0.001). In fact, more than 25% of patients with CD had normalized %CD3− values during follow-up (Figure 3), and the percentage of patients with CD and celiac lymphogram decreased from 80.8% to 65.4% at the final evaluation. Figure 4D shows an important overlap in the density of %CD3− in the final evaluation between the three study groups. Figure 5 shows the changes produced by a GFD in histological samples (pathology images) and in TCRγδ+ cells (gating strategy panels) in the three categories of patients analyzed: Marsh 3 CD, Marsh 1 CD, and NCGS.

## 4. Discussion

This is the first study assessing the diagnostic accuracy of the percentage of TCRγδ+ cells for CD and NCGS diagnosis in a large sample of patients assessed in a paired manner at baseline and long-term after GFD initiation. Because differentiating CD from NCGS after a GFD has been initiated is a challenging diagnostic situation, we included patients with NCGS as a disease control group.

The persistent clonal expansion of TCRγδ+ cells in CD has been previously documented by immunohistochemistry [10] and flow cytometry [8,9], showing that in most treated patients, the density of these cells remains elevated irrespective of the duration of a GFD [10]. The authors of these studies suggested further research be conducted to demonstrate the general clinical applicability of their findings [10]. In this sense, the present study offers new information regarding the use of flow cytometry, which is a well-standardized, highly reproducible, and affordable technique, for accurately quantifying the %TCRγδ+ subset in different clinical scenarios related to CD diagnosis. In addition, for the first time, we used a control group of healthy volunteers in whom CD and other disease states were strictly ruled out to establish the optimal cutoff point of %TCRγδ+ for CD diagnosis. This methodological aspect is important because most studies include patients with normal duodenal mucosa but with a variety of digestive symptoms as “healthy controls” [6,7,8,10,12,13,15,16,17]. This group of theoretically “healthy controls” may include patients with NCGS or potential CD, in whom TCRγδ+ values and the effect of a GFD are unknown. The selection of controls in the current study was strict in that only 12.6% of the total patients initially evaluated were included. This approach was used to rule out CD (none of the patients had permissive CD genetics) or many other disease conditions.

Using this group of healthy subjects as a “gold standard” control group, we identified the optimal cutoff point of isolated %TCRγδ+ for CD diagnosis at baseline before GFD initiation (>12.91) and under GFD (>13.31). With these values, we obtained a sensitivity > 0.85 and a specificity of 1 with an AUC of 0.95. For practical purposes, we suggest a cutoff point > 13%TCRγδ+ for CD diagnosis in any clinical situation, before or after starting a GFD, irrespective of its duration. This minimal change in the cutoff value compared to those obtained with the statistical methods we applied has little impact on diagnostic accuracy and instead facilitates its clinical applicability.

The celiac lymphogram has a lower diagnostic accuracy than the isolated assessment of %TCRγδ+, especially when a GFD has already been initiated. As mentioned, this is due to the progressive normalization of %CD3− values under a GFD. It is unclear if, with a longer follow-up period while patients remain on a strict GFD, the CD3− subset would eventually normalize in all patients. Therefore, the celiac lymphogram is mainly useful at the time of diagnosis with GCD, especially in doubtful cases. The cutoff point in this situation was a %TCRγδ+ > 8.64, which is very similar to the one previously obtained in our laboratory (%TCRγδ+ > 8.5) [8]. The advantage of combining the two subpopulations that conform to the celiac lymphogram (%TCRγδ+ >8.64 & %CD3− ≤16.3) is that the cutoff point for %TCRγδ+ is reduced while maintaining a similar diagnostic accuracy and specificity to that of the isolated evaluation of %TCRγδ+, which requires a cutoff point > 12.91 with a GCD.

Unlike patients with CD, patients with NCGS had a normal duodenal mucosa at baseline and after GFD, and in the present study, these patients were assessed with the same diagnostic work-up protocol and follow-up as patients with CD. The diagnostic accuracy of a cutoff value of %TCRγδ+ ≤ 13.31 applied to rule out CD in this group of NCGS patients was also very good. However, by applying the cutoff obtained for CD diagnosis in these patients while on a GCD, we found that 12 of them had a %TCRγδ+ > 13.31. We cannot rule out that some of these patients had potential CD, as suggested by the Oslo definition [31]. In fact, 5 out of 12 (41.7%) were first-degree relatives of patients with CD, and all of them, in contrast to healthy controls, had permissive CD genetics. Among these patients, the predominant gene was HLA-DQ8. Previous studies showed that patients with potential CD had low-to-moderate HLA-related risk more frequently than did those with high risk [32]. Therefore, the assessment of the percentage of TCRγδ+ cells in patients with a GFD and normal duodenal mucosa is very useful for the differential diagnosis of CD and NCGS. However, other complementary information, such as the clinical setting and genetics, should be accounted for to diagnose a few patients for whom doubts about the diagnosis remain, despite the valuable information provided by the %TCRγδ+.

Other proposed techniques for diagnosing CD in patients on a GFD include the detection of gut-homing CD8+ T cells in peripheral blood by flow cytometry after the reintroduction of 10 g gluten/d for 3 days [9,33], changes in plasma interleukin-2 after a single dose gluten challenge [34,35], and gluten-specific CD4 T-cell analysis with HLA-DQ2-gluten tetramers and IFN-γ enzyme-linked immune absorbent spot assay (ELISPOT) after 3–10 g of gluten/d for 3 days [34,36,37]. However, these tests are restricted to HLA-DQ2.5 patients. Formal comparisons between the different tests in clinical practice that could be complementary or performed in a sequential manner are warranted. We propose the assessment of isolated %TCRγδ+ cells as the first diagnostic approach for CD in patients who have already started a GFD due to its simplicity. In addition, there is no need for gluten challenge, which is usually not well accepted by patients.

TCRγδ+ cells have an immunoregulatory function and protective role in the mucosa against luminal microbes and antigens [38]. In the gut of active CD patients, effector TCRγδ+ IELs may predominate. In contrast, when gluten is withdrawn from the diet, TCRγδ+ IELs may become regulatory and may therefore contribute to recovery from gluten-driven epithelial damage [12,39]. This could explain why TCRγδ+ IELs remain elevated in patients on a GFD after the resolution of intestinal damage [10]. In contrast, CD3− cells, which are highly represented in healthy mucosa, are very reduced in active CD patients and tend to recover with mucosal healing [40]. The normalization of CD3− could explain why the diagnostic accuracy of the celiac lymphogram pattern decreases after the initiation of a GFD. In contrast, the accuracy of the %TCRγδ+ subset remained, irrespective of GFD duration, seroconversion, or mucosal healing.

Our study has strengths and limitations. The most important strengths are that this study assessed the accuracy of the CD diagnosis of subsets that conform to the intraepithelial lymphogram by flow cytometry, the use of a large sample size, an evaluation that involved before and after GFD, and the use of a group of true healthy controls as the gold standard of normality. In addition, for the first time, the long-term evolution of these cell subsets was evaluated in paired samples from patients with CD and from patients with NCGS receiving a GFD. The main limitation of our study is that there was selection bias in the group of patients with NCGS since only patients with two sequential biopsies were included. These patients underwent further biopsies as decided by the attending physician, and we cannot rule out that some patients diagnosed with NCGS in fact had seronegative potential CD. In any case, if all patients diagnosed with NCGS in our department during the study period, including patients with nonpermissive CD genetics, had been systematically biopsied under a GFD, the diagnostic accuracy of %TCRγδ+ applied to this patient group would likely have increased. Another limitation is that biopsy sampling was not scheduled at fixed follow-up times. Moreover, whether the cutoffs found are suitable for use in clinical practice should be evaluated externally. This validation is anticipated to be performed soon.

TCRγδ+ cells are a very useful biomarker for CD diagnosis in patients on a GFD because the increased percentages are maintained irrespective of the diet. This biomarker is also useful in cases of doubtful CD (patients with seronegative atrophy and patients with mild enteropathy seropositive or not). Increased percentages of TCRγδ+ strongly suggest CD, being an essential supportive method. Patients with NCGS have normal percentages of TCRγδ+ cells. Therefore, normal values of this subpopulation exclude the diagnosis of CD in a patient having an unequivocal and sustained clinical improvement with a GFD. The exclusion of CD in patients with NCGS is a part of the diagnostic work-up.

## 5. Conclusions

In summary, we demonstrated that the assessment of the percentage of TCRγδ+ cells in the duodenal mucosa using flow cytometry has good diagnostic accuracy for CD diagnosis in patients receiving a GFD and is therefore useful for diagnosing patients for whom the basal standard diagnostic approach was not used. We propose evaluating γδ T cells by flow cytometry in routine clinical practice for diagnosing either CD or NCGS in patients on a GFD.

## Figures and Tables

**Table 1:** Baseline characteristics of patients with celiac disease, non-celiac gluten sensitivity, and healthy controls.

Variables	Marsh 3 Celiac Disease (n = 89)	Seropositive Marsh 1 Celiac Disease (n = 15)	Marsh 0 Non-Celiac Gluten Sensitivity (n = 37)	Marsh 0 Healthy Individuals (n = 18)
Age (years) ^a^	34.00 [19.00; 44.00]	37.00 [21.00; 53.00]	41.00 [35.00; 46.00]	25.00 [22.25; 26.75]
Female (n, %)	69 (77.53%)	10 (66.67%)	30 (81.08%)	12 (66.67%)
Duration of GFD to 2nd biopsy (years) ^a^	2.00 [2.00; 3.00]	2.00 [1.50; 2.50]	2.00 [1.00; 4.00]	NA [NA; NA]
Celiac serology (n, %)				
Positive anti-tTG2 ^b^	85 (95.51%)	15 (100%)	0 (0.00%)	0 (0.00%)
Negative anti-tTG2	4 (4.49%)	0 (0.00%)	37 (100.00%)	18 (100.00%)
%TCRγδ+ cells ^a^	22.65 [16.90; 32.64]	25.42 [13.70; 35.75]	9.43 [4.10; 14.66]	5.75 [1.80; 8.59]
%CD3− cells ^a^	2.78 [1.66; 5.00]	6.41 [2.44; 10.96]	16.56 [8.65; 28.16]	21.97 [16.78; 26.40]
%IEL/100 epithelial cells ^a^	50.00 [40.00; 63.00]	35.00 [29.37; 47.00]	19.00 [13.00; 22.00]	14.50 [13.00; 17.75]
HLA-DQ genotype (n, %)				
HLA-DQ2.5	71 (86.59%)	15 (100.00%)	15 (40.54%)	0 (0.00%)
HLA-DQ8	5 (6.10%)	0 (0.00%)	16 (43.24%)	0 (0.00%)
HLA-DQ2.2	6 (7.32%)	0 (0.00%)	4 (10.81%)	0 (0.00%)
HLA-DQ7.5	0 (0.00%)	0 (0.00%)	1 (2.70%)	0 (0.00%)
All negative	0 (0.00%)	0 (0.00%)	1 (2.70%)	18 (100%)

^a^ Median (interquartile range: 25%; 75%). ^b^ Borderline values were considered positive if confirmed by positive serum IgA anti-endomysium antibodies. Abbreviations: GFD: gluten-free diet; HLA: human leukocyte antigen; TCR: T-cell receptor; IEL: intraepithelial lymphocytes; NA: not applicable.

**Table 2:** Evolution of the variables before (baseline) and after a gluten-free diet (final).

	Marsh 3 Celiac Disease			Seropositive Marsh 1 Celiac Disease			Marsh 0 Non-Celiac Gluten Sensitivity		
Variables	Baseline (n = 89)	Final (n = 89)	*p* Value	Baseline (n = 15)	Final (n = 15)	*p* Value	Baseline (n = 37)	Final (n = 37)	*p* Value
Histology (n, %)									
Marsh 0	0 (0.00%)	31 (34.83%)		0 (0.00%)	14 (93.33%)		37 (100.00%)	37 (100.00%)	
Marsh 1	0 (0.00%)	42 (47.19%)		15 (100.00%)	1 (6.67%)		0 (0.00%)	0 (0.00%)	
Marsh 3	89 (100.00%)	16 (17.98%)		0 (0.00%)	0 (0.00%)		0 (0.00%)	0 (0.00%)	
%IEL/100 epithelial cells ^a^	NA [NA; NA]	NA [NA; NA]		35.00 [29.37; 47.00]	22.00 [20.50; 24.70]	<0.001	19.00 [13.00; 22.00]	18.00 [13.40; 21.00]	0.475
Celiac serology (n, %)									
Positive Anti-tTG2 ^b^	85 (95.51%)	19 (22.10%)		15 (100%)	2 (14.28%)		0 (0.00%)	0 (0.00%)	
IEL flow cytometry ^a^									
%TCRγδ+ cells ^a^	22.65 [16.90; 32.64]	26.99 [17.40; 36.69]	0.020 ^c^	25.42 [13.70; 35.75]	25.17 [18.91; 34.66]	>0.999 ^c^	9.43 [4.10; 14.66]	6.40 [3.20; 11.00]	0.022 ^c^
%CD3− cells ^a^	2.78 [1.66; 5.00]	4.80 [2.50; 13.28]	<0.001 ^c^	6.41 [2.44; 10.96]	11.20 [3.98; 14.33]	0.003 ^c^	16.56 [8.65; 28.16]	19.37 [8.80; 30.53]	0.200 ^c^

^a^ Median (interquartile range: 25%; 75%). ^b^ Borderline values were considered positive if confirmed by positive serum IgA anti-endomysium antibodies. ^c^ Paired Wilcoxon signed rank test. Abbreviations: GFD: gluten-free diet; TCR: T-cell receptor; IEL: intraepithelial lymphocytes; anti-tTG2: IgA-tissue transglutaminase antibody; NA: not applicable.

**Table 3:** Exploration of the best cutoff value for separating TCRγδ+ IELs and celiac lymphogram values between CD patients and healthy individuals (gold standard) via two methods (the Youden index and a logistic model).

	Isolated Increase TCRγδ+		Celiac Lymphogram ^a^	
Best Cutoff Value	Youden Index	Logistic Model	Youden Index	Logistic Model
Diagnostic accuracy with GCD (baseline)	%TCRγδ+ > 12.91	%TCRγδ+ > 8.64	%TCRγδ+ > 12.91 and %CD3− ≤ 13.33	%TCRγδ+ > 8.64 and %CD3− ≤ 16.3
Sensitivity	0.87 [0.78, 0.92]	0.95 [0.89, 0.98]	0.82 [0.73, 0.88]	0.91 [0.84, 0.96]
Specificity	1 [0.78, 1]	0.78 [0.52, 0.93]	1 [0.78, 1]	0.94 [0.71, 1]
PPV	1 [0.95, 1]	0.96 [0.90, 0.99]	1 [0.95, 1]	0.99 [0.94, 1.00]
NPV	0.56 [0.38, 0.73]	0.74 [0.49, 0.90]	0.49 [0.32, 0.65]	0.65 [0.44, 0.82]
Accuracy	0.89 [0.81, 0.93]	0.93 [0.86, 0.96]	0.84 [0.77, 0.90]	0.92 [0.85, 0.96]
Diagnostic accuracy with GFD (final)	%TCRγδ+ > 13.31	%TCRγδ+ > 8.67	%TCRγδ+ > 13.31 and %CD3− ≤ 13.33	%TCRγδ+ > 8.67 and %CD3− ≤ 16.6
Sensitivity	0.86 [0.77, 0.91]	0.92 [0.86, 0.96]	0.65 [0.55, 0.74]	0.81 [0.72, 0.88]
Specificity	1 [0.78, 1]	0.78 [0.52, 0.93]	1 [0.78, 1]	0.94 [0.71, 1.00]
PPV	1 [0.95, 1]	0.96 [0.89, 0.99]	1 [0.93, 1]	0.99 [0.93, 1.00]
NPV	0.55 [0.37, 0.72]	0.64 [0.41, 0.82]	0.33 [0.22, 0.48]	0.46 [0.30, 0.63]
Accuracy	0.88 [0.80, 0.93]	0.90 [0.83, 0.95]	0.70 [0.61, 0.78]	0.83 [0.75, 0.89]

^a^ Celiac lymphogram: increase TCRγδ+ plus decrease CD3− cells. Abbreviations: IEL: intraepithelial lymphocytes; TCR: T-cell receptor; GFD: gluten-free diet; GCD: gluten-containing diet; PPV: positive predictive value; NPV: negative predictive value.

**Table 4:** Characteristics of patients with non-celiac gluten sensitivity (all Marsh 0 [<25 IELs] with negative celiac serology) and %TCRγδ+ > 13.31.

Age	Gender	Main Clinical Feature	First-Degree Relatives with CD	HLA-DQ Genotype	Baseline %TCRγδ+ Cells	Final %TCRγδ+ Cells
67	Female	Bloating	No	DQ8	13.51	7.50
30	Female	Abdominal pain	Yes	DQ2.5	14.29	11.50
39	Female	Diarrhea	Yes	DQ8	14.66	11.00
64	Female	Dyspepsia	No	DQ2.5	15.22	7.73
63	Female	Diarrhea	No	DQ8	16.36	1.88
39	Female	Bloating	No	DQ8	18.16	19.52
41	Female	Bloating	Yes	DQ8	18.34	14.86
36	Female	Dyspepsia	Unknown	DQ2.2	18.73	28.83
60	Female	Bloating	No	DQ2.5 and DQ8	19.00	27.40
51	Female	Bloating	Yes	DQ8	21.90	15.80
35	Female	Dyspepsia	No	DQ2.5	25.94	20.00
63	Male	Diarrhea	Yes	DQ8	37.51	29.79
46	Female	Diarrhea	No	DQ8	11.04	15.48

Abbreviations: TCR: T-cell receptor; HLA: human leukocyte antigen; CD: celiac disease.

## Data Availability

The database underlying this article is available upon reasonable request.

## Supplementary Materials

The following supporting information can be downloaded at: https://www.mdpi.com/article/10.3390/nu16142294/s1; Supplementary Table S1: Dyspepsia test for healthy volunteers. Supplementary Table S2: Analytical study of healthy volunteers.
