# Supplementary material for: γδ+ T-Cells Is a Useful Biomarker for the Differential Diagnosis between Celiac Disease and Non-Celiac Gluten Sensitivity in Patients under Gluten Free Diet
Source: Nutrients. 2024 Jul 17;16(14):2294. doi: 10.3390/nu16142294 (PMC11279444; doi:10.3390/nu16142294)
Supplement: Supplementary file 1 [file nutrients-16-02294-s001.zip › nutrients-3087040-supplementary.pdf]

Supplementary Materials:

Supplementary Table S1. Dyspepsia test for healthy volunteers.

**Dyspepsia test**
**Medical history:**
1. Active smoker (anyone who has smoked any number of cigarettes daily for the past month)<br>☐ Yes ☐ No<br>If yes, please specify the number of cigarettes smoked per day and/or week:<br>2. Alcohol consumption:<br>* A standard drink contains approximately 10g of alcohol (in Europe). Examples of standard drinks include: 330 ml of 5% beer, 140 ml of 12% wine, 90 ml of fortified wines (e.g., sherry) at 18%, 70 ml of 25% liqueur or aperitif, 40 ml of 40% spirits.<br>* Risky consumption: The World Health Organization (WHO) defines this as the regular daily consumption of 20 to 40g of alcohol for women, and 40 to 60g of alcohol for men.<br>☐ Yes ☐ No<br>3. Relevant medical history:<br>☐ Yes ☐ No<br>If the answer is yes, please specify:<br>4. Do you have a 1st or 2nd degree family history of celiac disease?<br>☐ Yes ☐ No<br>5. Do you have a 1st or 2nd degree family history of inflammatory bowel disease?<br>☐ Yes ☐ No<br>6. Do you have a personal history of neoplasms or inflammatory diseases?<br>☐ Yes ☐ No<br>7. Do you have a history of surgeries?<br>☐ Yes ☐ No<br>If the answer is yes, please specify:
**Usual medication:**
8. Do you take any medication regularly?<br>☐ Yes ☐ No<br>If the answer is yes, please specify:<br>9. Have you used non-steroidal anti-inflammatory drugs (NSAIDs) in the last 4 weeks?<br>☐ Yes ☐ No<br>If the answer is yes, specify:
**Current clinical status:**
10. Usual bowel habits: Number of bowel movements and their consistency (hard, shaped, soft/pasty, or liquid)?"<br>11. Have you experienced any digestive issues that required consultation with a doctor in the last 6 months?<br>☐ Yes ☐ No

12. Do you experience abdominal distension more than once a month?

☐ Yes ☐ No

13. Do you experience diarrhoea more than once a month?

☐ Yes ☐ No

14. Do you experience constipation more than once a month?

☐ Yes ☐ No

15. Have you experienced bloody diarrhoea in the last 6 months?

☐ Yes ☐ No

16. Have you experienced gastroenteritis in the last 6 months?

☐ Yes ☐ No

17. Do you experience stomach or abdominal pain more than once a month?

☐ Yes ☐ No

18. Have you experienced unintentional weight loss in the last 6 months?

☐ Yes ☐ No

If the answer is yes, please specify:

19. Have you experienced gastrointestinal bleeding at any time in the last 6 months?

☐ Yes ☐ No

20. Have you experienced clinical symptoms such as progressive difficulty or pain in swallowing, heartburn, persistent vomiting, or jaundice (yellowing of the skin) in the last 6 months?

☐ Yes ☐ No

21. Have you experienced unexplained iron deficiency anaemia in the last 6 months?

☐ Yes ☐ No

22. Do you regularly follow a varied Mediterranean diet that includes gluten?

☐ Yes ☐ No

If the answer is negative, please specify:

23. Is there a possibility of pregnancy at the present time?

☐ Yes ☐ No

24. Have you travelled to tropical countries in the last 6 months?

☐ Yes ☐ No

If the answer is negative, please specify:

Supplementary Table S2. Analytical study of healthy volunteers.

Analytical study	
1. Haemoglobin<br>☐ g/dL<br>2. Platelets<br>☐ x10^9/L<br>3. Leukocytes<br>☐ x10^9/L<br>4. Neutrophils<br>☐ %<br>5. Alanine aminotransferase (ALT)<br>☐ U/L<br>6. Gamma-glutamyl transferase (GGT)<br>☐ U/L<br>7. Alkaline phosphatase (ALP)<br>☐ U/L<br>8. Creatinine<br>☐ mg/dL<br>9. Urea<br>☐ mg/dL	10. Glomerular filtration rate<br>☐ mL/min/1.73m^2.<br>11. Prothrombin Time (PT) and International Normalized Ratio (INR)<br>☐ % ☐<br>12. IgA-tissue transglutaminase antibodies<br>☐ U/mL<br>13. Presence of the HLA-DQ2.5<br>☐ Positive ☐ Negative<br>14. Presence of the HLA-DQ8<br>☐ Positive ☐ Negative<br>15. Presence of the HLA-DQ2.2<br>☐ Positive ☐ Negative<br>16. Presence of the HLA-DQ7.5<br>☐ Positive ☐ Negative<br>17. *Helicobacter pylori* serology:<br>☐ Positive ☐ Negative
